# Knowledge, Attitude, and Perceptions of Pharmacists and Pharmacy Students towards Pharmacogenomics in Zimbabwe

**DOI:** 10.3390/pharmacy5030036

**Published:** 2017-06-30

**Authors:** Nyasha Muzoriana, Samuel Gavi, Victoria Nembaware, Milcah Dhoro, Alice Matimba

**Affiliations:** 1 School of Pharmacy, College of Health Sciences, University of Zimbabwe, P.O. Box A178, Avondale, Harare, Zimbabwe; nyashamuzoriana@gmail.com; 2 Department of Clinical Pharmacology, College of Health Sciences, University of Zimbabwe, P.O. Box A178, Avondale, Harare, Zimbabwe; samuelgavi@yahoo.com (S.G.); milcah_dhoro@yahoo.com (M.D.); 3 Computational Biology Group, Department of Integrative Biomedical Sciences, Institute of Infectious Disease and Molecular Medicine, Faculty of Health Sciences, University of Cape Town, Anzio Road, Observatory 7925 Cape Town, South Africa; vnembaware@gmail.com

**Keywords:** pharmacogenomics, attitude, knowledge, perception, education

## Abstract

The potential of pharmacogenomics (PGx) to positively impact health outcomes and quality of healthcare is well-established. However, the application of available evidence into clinical practice is still limited due to limited knowledge among healthcare professionals, including pharmacists. As a start towards building capacity for PGx education, we assessed knowledge, attitudes, and perceptions about PGx among practising pharmacists and pharmacy students. A cross-sectional study was conducted among pharmacists and undergraduate pharmacy students selected using a convenient sampling method—a 37-question survey instrument was used to obtain information regarding PGx among the participants. Out of a total of 131 participants, 56% of respondents showed fair-to-good PGx knowledge. Respondents’ self-reported assessment indicated that 88% had average and above knowledge scores in PGx. Practising pharmacists in Zimbabwe have positive attitudes towards PGx and would support its application to improve treatments. However, there were concerns about security and discrimination when genomics data is used by those who do not understand its meaning. Participants agreed that they would play a leading role in PGx testing if provided with appropriate training. The interest in PGx is challenged by their limited knowledge and understanding of genetics, suggesting a need to update curricula for pharmacy students and for continuing health education programmes.

## 1. Introduction

Pharmacogenomics combines pharmacology (the science of drugs) and genomics (the study of genes and their functions) to develop safe and efficacious medications tailored to a person’s genetic makeup [1]. Pharmacogenomics contributes towards personalised and precision medicine which aims to provide the right drug at the right dose for the right indication at the right time using the right route. While pharmacogenetics refers to the association of single to a few genetic markers with drug responses, pharmacogenomics is more encompassing of current and future application of large-scale genome analysis, and both terms may be used interchangeably and will be referred to as PGx. Advances in rapid testing technologies and the development of pre-emptive genotyping programs suggest that personalized medicine through PGx is a feasible reality [2]. The success of PGx translation depends on its practical application by clinicians and pharmacists. Therefore, if appropriately applied, PGx has the potential to substantially change health practices and the expectations of health professionals, governments, and consumers.

PGx presents the most promising application of human genomics due to the larger effect sizes of PGx variants compared to other complex traits [3]. To date, over 100 medications include PGx information which can be used for guiding treatment [4]. Despite the established contribution of genetic variations to drug responses, there remains a slow uptake in clinical application due to limited knowledge and awareness about genomics among health professionals [5,6]. Therefore, as PGx applications become more established, education of health professionals is paramount both at the undergraduate level and in continuing health education. Due to their extensive knowledge in pharmacology and pharmacotherapy, pharmacists are ideally placed to play an essential role for the clinical implementation of PGx [5,7,8].

In low- and middle-income countries (LMICs) such as Zimbabwe, limited resources and poor understanding of PGx remains a challenge. In addition, the scarcity of relevant population data—particularly in Africa—means that basic and translational research are required to tailor recommendations for African people [9]. However, in some cases, despite availability of population specific evidence supporting the use of PGx information in the use of some drugs such as efavirenz [9], there are inadequate testing facilities and healthcare teams are not well-equipped with knowledge and resources to incorporate into practice.

PGx training among pharmacists in developing countries will support the development of PGx testing in the future. Although several pharmacy training programmes implement PGx in their curricula in USA and Europe [7,10,11,12], developing countries are lagging behind in updating curricula to incorporate such emerging topics in health education. As the availability of resources continues to improve and genomics in Africa takes centre-stage [13,14,15], it is important to understand the current status and needs for PGx education in order to develop appropriate training programmes among pharmacists and other health professionals.

The University of Zimbabwe, College of Health Sciences is the largest health training institution in the country, training pharmacists, doctors, nurses, and other health professionals. The undergraduate pharmacy degree currently implements an introductory one-hour session in PGx. PGx is also taught over a few hours in postgraduate programmes in pharmacology and medicine. In comparison to USA and other universities, this is not adequate [7,10,11].

We set out to understand the level of knowledge, attitudes, and practices among pharmacists and pharmacy students in Zimbabwe and to determine educational needs for PGx education in Zimbabwe. This will identify gaps in awareness and for building capacity for PGx training through the development of curricula for undergraduate and postgraduate students and pharmacists.

## 2. Methods

Ethical approval for the study was granted by the University of Zimbabwe-College of Health Sciences (UZ-CHS) institutional review board (JREC), reference Number: 355/14, and the Medical Research Council of Zimbabwe (MRCZ), reference Number: MCRZ/B/758. The Pharmacist Council of Zimbabwe gave permission to interview pharmacists.

A questionnaire-based cross-sectional study was conducted in Harare, the capital city of Zimbabwe. The target populations were practicing pharmacists in Zimbabwe and final year pharmacy students from the UZ-CHS, which is the largest training institution for health professionals in the country. Practicing pharmacists were recruited from participants of continuing education meetings (CEs) held at UZ-CHS. These included representatives from the UZ-CHS School of Pharmacy, retail, and hospital pharmacies. Final year students were given an open invitation to participate, and everyone who consented was recruited. Upon request, an electronic questionnaire was emailed to study participants who were not present during the CEs. The questionnaire was adopted from a similar study that was done in Malaysia by Bannur Z et al. [16], and was comprised of four sections: demographics (six questions), knowledge (six questions), attitudes (eight questions), and perceptions (seventeen questions).

Respondent characteristics included gender, age, practice setting, highest education, and years of practice. Level of knowledge was measured on a scale of 0–5, 0 being the lowest score and 5 the highest score; Cronbach’s alpha for this section was 0.715. Self-reported assessment of their knowledge in PGx was categorised into “very weak”, “weak”, “average”, “above average”, “good”, and “very good”. Participants’ attitudes towards the likely impact of PGx was categorised into “likely”, “neutral”, and “unlikely”. Participants’ concerns towards PGx applications was characterised according to “not concerned”, “somewhat concerned”, and “very concerned”. Participants’ attitudes towards PGx applications was categorised as “very uncomfortable”, “uncomfortable”, and “comfortable”. Perceptions of pharmacists and pharmacy students towards PGx were categorised according to “disagree”, “neutral”, and agree”. Cronbach’s alpha for this section was calculated to be 0.886—a valid indicator of the reliability and consistency of the testing instrument. Data collected through the survey tool was entered into EpiData Software, and analysis was done using STATA-14.

## 3. Results

### 3.1. Characteristics of Study Participants

The study response rate was 82% (131/160 questionnaires were completed), which included 45 final year students and 86 qualified pharmacists. The demographic information of the study participants is summarized in Table 1. Most of the pharmacists were male (53%) and 30 years of age and above (65%). The students were mostly male (62%) and less than 30 years old (86%). The mean and median time for practising among pharmacists was 8 and 6 years, respectively (sd = 6.1; range 1–30 years). Final year students reported having been attached to an academic setting (94%) and without any practising experience, except for two having been attached to a hospital and industry settings. The majority of pharmacists’ practise within retail (60%), followed by 22% practising in hospitals (public and private). Amongst the pharmacists, 19% had a Master’s degree and 78% had a Bachelor’s degree.

### 3.2. PGx Knowledge of Study Participants

A total of 16 participants (12%) had a score ≥4 out of 5 for the assessment of knowledge on PGx, which showed an above-average understanding of the subject; the remaining 115 participants (88%) had a score ≤3 out of 5 showing an average to below-average understanding of the subject. A univariate regression analysis showed no statistically significant difference in PGx knowledge scores between pharmacists and final year pharmacy students (β = 0.13; *p*-value = 0.4). Table 2 shows that most participants understood that subtle differences in a person’s genome can affect drug response (97%) and that genetic determinants do not change over a person’s lifetime (63%). Regarding whether the warfarin package insert includes a warning, only 26% answered correctly based on the currently available brands available in Zimbabwe.

**Figure 1:**
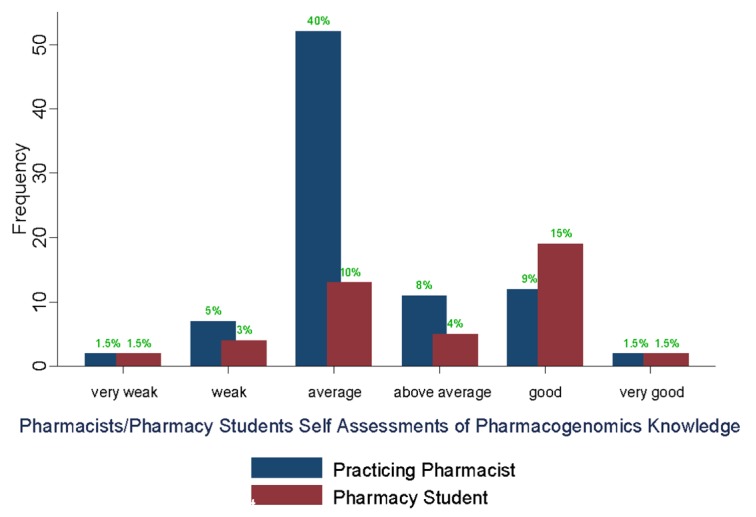
Pharmacist/pharmacy student self-assessment of PGx knowledge.

The study participants’ self-reported assessment of their knowledge in PGx showed that 80 participants (61%) rated themselves as being average-to-weak and very weak (Figure 1). A univariate logistic regression analysis showed pharmacists rating their knowledge lower than final year pharmacy students (OR = 0.3, *p*-value = 0.002).

### 3.3. Study Participants’ Attitudes Towards PGx Applications

We aimed to understand the participants’ attitudes towards the impact of PGx applications. We retained the use of warfarin as an established example with potential application of PGx in clinical practice. Table 3 shows that the majority of participants indicated that it was likely that PGx testing would reduce the time it takes to find the optimal dose for patients on warfarin (83%), as well as reduce related adverse reactions. In addition, participants indicated that it was likely that PGx could reduce the cost of new drug development (62%). In an ordinal regression analysis (Table 3), Pharmacists had a more positive attitude regarding the use of PGx to reduce time for optimising warfarin dose (OR = 3.0; *p*-value = 0.02).

Eighty percent (80%) of participants indicated that they would be comfortable with the incorporation of PGx in warfarin dosing for patients. Most of the study participants further reported that if they themselves were patients requiring warfarin, they (81%) would be comfortable with PGx testing to guide dosing.

Ninety-two percent of respondents were concerned that unauthorised persons may gain access to a patient’s PGx results. The majority of respondents were concerned about the possibility of PGx data being used to discriminate against patients in employment, and by insurance companies (92%). There were no significant differences in these aspects between pharmacists and pharmacy students.

### 3.4. Perceptions of Study Participants Towards PGx

Respondents highlighted the lack of training in current curricula with the pharmacists more likely to indicate that PGx was not taught in pharmacy school (*p* < 0.0005). Most study participants agree that PGx is important (88%), that pharmacists should be knowledgeable in the subject (94%), and that PGx should be taught in pharmacy school (85%) as well as incorporated in continuing health professional education (84%) (Table 4). Participants agree that if taught PGx, they would be able to be consulted (94%), provide advice about PGx (87%), and identify medicines requiring PGx testing (88%). Some participants indicate that they were familiar with PGx sources (67%) and tests in Zimbabwe (48%). Most study participants (94%) agree that pharmacists should be knowledgeable about PGx, that this field is relevant to their current practice (74%), and that pharmacists must recommend PGx in practice (76%).

Participants agreed that PGx could control medical costs (83%) and that it should was relevant in clinical practice (74%). Participants agreed that that in future, pharmacists should be consulted for PGx testing (79%) and when using genetic information for adjusting treatment (71%). In comparison to the pharmacists in training, qualified pharmacists were more in agreement that pharmacists should carry out PGx tests in the future (OR = 6.8; *p* < 0.0005). Most of the participants (93.9%) reported that it was their role to counsel patients and provide information regarding PGx tests.

## 4. Discussion

Pharmacists need to adjust their roles in clinical practice so that they become more patient-oriented to enhance implementation of personalized pharmacotherapy services [17]. By integrating PGx and pharmaceutical care, the pharmacist becomes a key player in the translation of PGx into clinical practice and personalized medicine.

Pharmacists in Zimbabwe have limited-to-fair knowledge and awareness of the PGx as highlighted in this study—a scenario not unique to Zimbabwe only or pharmacists but is a global concern across most healthcare professionals as shown by various studies in other LMICs whereby below-average knowledge of PGx was noted [14,18,19]. As a result, in Ghana PGx is now taught in some training institutions and as continuing education for doctors and pharmacists [14]. Similarly, in a cross-sectional study done in Malaysia among physicians and pharmacists, knowledge was low but interest was very high, and participants preferred to learn via continuous professional education [16].

This study showed that pharmacists in Zimbabwe are keen to see PGx being implemented as part of clinical care. Several studies have shown the same level of anticipation among pharmacists having a positive attitude towards PGx and willing to incorporate PGx into practice [18,20,21,22,23,24]. In this study, pharmacists had a positive attitude towards PGx applications. However, ethical concerns included privacy issues and discrimination. In addition, where a patient carries markers which make them more susceptible to life-threatening events, and being in a country where alternative medicines may not be available, it would be important to consider how such issues would be considered as current guidelines and resources do not address these issues in an African-specific context. Multi-stakeholder consultation is required to understand the potential impact of PGx testing in the clinic in order to develop representative policies and laws.

Results from this study emphasise that PGx should be an integral part of the healthcare system in Zimbabwe and that Pharmacists should be consultants in PGx for other healthcare providers. Studies conducted in Malaysia and Nigeria reported similar results, although hurdles such as the economic benefit, ethical concerns, and training were highlighted [14,15]. The positive perceptions and attitudes demonstrated by this study may indicate that PGx has the potential to be implemented by health professionals, and pharmacists in particular.

Regardless of the type of educational level and practice setting, all pharmacists had positive attitudes and perceptions towards PGx. However, the pharmacists’ lack of knowledge and confidence towards implementation of the PGx highlights the need for targeted PGx education. PGx education programmes have been successfully implemented for health professionals in other countries [11,25,26,27], and could provide a basis for the development of targeted programmes locally. Zgheib et al. reported on the experiences of PGx teaching in low- and middle-income countries [28], while in Africa studies have focused on understanding knowledge and attitudes [14,15]. PGx education may be further challenged by lack of PGx expertise and lack of practice setting for practical training [8]. Train-the-trainer programs can be implemented to alleviate this gap [12,29]. In addition, instructional software has been used to improve understanding and potential application of PGx, and could be applied for online training modules [30].

Pharmacists indicated an absence of PGx education during their training, suggesting the need for updating the current curriculum to incorporate PGx more comprehensively. Continuing health education programmes could be implemented for pharmacists who are already practicing. In the meantime, further research among African populations is required to tailor the curricula with locally relevant genetic information. Few studies to-date have conducted comprehensive analysis of PGx among African populations, revealing the high level of genomic diversity in pharmacologically important genes [13]. However, initiatives for building capacity for genomics in Africa provide a stepping stone towards research and clinical translation within the African environmental and population-specific context [14,22,31]. While several programmes exist for developing the capacity of PGx, in Africa, the Human, Heredity and Health in Africa (H3Africa) initiative has taken centre-stage in analysing and interpreting genomics data from a wide range of projects across various disease areas.

Such initiatives, combined with other existing international programmes, databases will allow improved understanding of genomics and drug response. To this end, the Clinical PGx Implementation Consortium (CPIC) supported by the PharmGKB and other databases created a database aimed at guiding clinicians to make patient care decisions for specific drugs based on numerous factors, including PGx information; the database is being reviewed and updated as more evidence emerges [32]. Despite these advances, there remains limited awareness and knowledge and awareness of PGx in developing countries in Africa such as Zimbabwe. Therefore, training, education, and other resources such as genetic testing facilities, counselling, and information databases are needed to support the application of PGx and to provide appropriate care to patients [31].

The provision of healthcare services is a multi-stakeholder process; thus, educating the pharmacist alone would not suffice, as poor knowledge levels have been reported from research conducted across different healthcare professions. There should be an adequate appreciation of PGx by other healthcare team players such as doctors and nurses. In the US, the National Coalition for Health Professional Education in Genetics (NCHPEG) made recommendations on “Core Competencies in Genetics Essential for All Health-Care Professionals” [33]. This resulted in the crafting of competence standards for nurses [34], with most pharmacy schools now including PGx in their curricula [1] after the American Council on Pharmacy Accreditation set PGx as a requirement in the pharmacy curriculum in 2007 [35]. The African Genomic Medicine Training Initiative is currently working towards building such core competencies for healthcare professionals in Africa.

## 5. Conclusions

Due to the emerging genomic technologies and their potential for clinical use, there is need to address the knowledge gap for PGx by training pharmacists, and further tailoring for the local context is required. Such training could be either through professional development courses or through the integration of PGx into the curriculum for pharmacy students. The survey brought out a general agreement that the pharmacists should be required to have knowledge of PGx in order to be able to recommend PGx testing within their practice, accurately apply the results of PGx tests to drug therapy selection, dosing or monitoring, (and understand ethical issues in PGx testing. This will enable pharmacists to play an active role in shaping the future of PGx in Zimbabwe and other LMICs where similar challenges in limited resources and expertise are currently faced.

## Figures and Tables

**Table 1:** Characteristics of study participants.

Characteristics	Pharmacists	Final Year Pharmacy	
	N = 86 (66%)	N = 45 (34%)	
Gender			
Male	46 (53%)	28 (62%)	Fischer’s Exact = 0.5
Female	37 (43%)	16 (36%)	*p*-value = 0.2
Missing Values*	*3 (4%)*	*1 (2%)*	
Age (years)			Mean = 30.5 years
			Median = 30.0 years
<30 years	14 (16%)	39 (86%)	Fischer’s Exact ≤0.0005
≥30 years	56 (65%)	1 (2.0%)	*p*-value ≤0.0005
Missing Values*	*16 (19%)*	*5 (11%)*	
Practice Setting			Fischer’s Exact ≤0.0005 *p*-value ≤0.0005
Retail	52 (60%)	0 (0%)	
Hospital	18 (22%)	1 (2%)	
Academia	10 (12%)	42 (94%)	
Industry	2 (2%)	1 (2%)	
Regulatory	1 (1%)	0 (0%)	
Missing Values*	*3 (3%)*	*1 (2%)*	
Years of Pharmacy Practice		-	Mean = 7.8 years s.d = 6.1 years
1–5 years	39 (45%)		
6–10 years	32 (37%)		
11–15 years	7 (8%)		
16–20 years	3 (4%)		
21–25 years	2 (2%)		
26–30 years	3 (4%)		
Highest Education			Fischer’s Exact ≤0.0005 *p*-value ≤0.0005
Bachelor’s degree	67 (78%)	2 (4%)	
Master’s Degree	16 (19%)	0 (0%)	
PhD/D. Phil	3 (3%)	0 (0%)	
High School	0 (0%)	42 (94%)	
Missing Values*		*1 (2%)*	

Missing Values*: Denote number of participants who did not answerr certain questions. Missing values were not included in final analysis.

**Table 2:** Participant’s knowledge of pharmacogenomics (PGx).

Statement	True % (N = 131)	False % (N = 131)	Not Sure % (N = 131)
Subtle differences in a person’s genome can have a major impact on how the person responds to medications.	127 (97%) *	1 (1%)	3 (2%)
Genetic determinants of drugs response change over a person’s lifetime.	30 (23%)	83 (63%) *	18 (14%)
Genetic variants can account for 95% of the variability in drug disposition and effects.	18 (14%) *	59 (45%)	54 (41%)
The package insert for warfarin includes a warning about altered metabolism in individuals who have specific genetic variants.	34 (26%)	34 (26%) *	63 (48%)
PGx testing is currently available for most medications.	8 (6%)	86 (66%) *	37 (28%)

**Answers to Knowledge Questions: 1.** True **2.** False **3.** True **4.** True **5.** False; * Percentage of respondents who got the questions correct on each knowledge question.

**Table 3:** Participants’ attitudes towards pharmacogenomics (PGx) applications by profession.

Question/Statement	Response N = 131 (%) Ordinal Logistic Regression Output					
	Unlikely	Neutral	Likely	O.R	S.E	*p*-Value
How likely is it that PGx testing will help to decrease the number of adverse drug reactions?	13 (10%)	13 (10%)	105 (80%)	0.4	0.2	0.06
How likely is it that PGx testing will help to decrease the cost of developing new drugs?	32 (24%)	18 (14%)	81 (62%)	1.9	0.7	0.06
How likely is it that PGx testing will reduce the time it takes to find the optimal dose for patients on warfarin?	6 (5%)	16 (12%)	109 (83%)	3.0	1.4	0.02
How likely is it that PGx testing will help reduce ADR’s due to warfarin?	4 (3%)	20 (15%)	107 (82%)	0.6	0.3	0.3

**Table 4:** Perceptions of pharmacists and pharmacy students towards PGx.

Question	Responses (N = 131) Ordinal Logistic Regression Output					
	Disagree	Neutral	Agree	O.R	S.E	*p*-Value
PGx was taught in Pharmacy School	54 (41%)	17 (13%)	60 (46%)	0.1	0.05	<0.0005
PGx is an important field in Pharmacy	7 (5%)	9 (7%)	115 (88%)	1.5	1.0	0.6
Pharmacists must know PGx	3 (2%)	5 (4%)	123 (94%)	6.6	35.7	0.7
PGx should be added to Pharmacy School	8 (6%)	12 (9%)	111 (85%)	2.6	1.6	0.1
PGx should be taught in Pharmacy Continuing Education Seminars	1 (1%)	20 (15%)	110 (84%)	0.9	05	0.9
I should be able to consult if taught PGx	0 (0%)	8 (6%)	123 (94%)	2	6.4	0.8
If trained, I can advise of therapy changes after PGx testing	0 (0%)	18 (14%)	113 (87%)	0.5	1.1	0.8
Training will help Pharmacists identify medicines requiring PGx testing	2 (1%)	14 (11%)	115 (88%)	1.6	0.9	0.4
I know reliable sources of info on PGx	6 (5%)	37 (28%)	88 (67%)	1.3	0.5	0.5
I know PGx tests used in Zimbabwe	30 (23%)	38 (29%)	63 (48%)	0.9	0.3	0.7
PGx test will control medicine costs	6 (5%)	16 (12%)	109 (83%)	0.7	0.6	0.7
PGx is relevant to my current practice	3 (2%)	31 (24%)	97 (74%)	0.7	0.3	0.5
Pharmacists must recommend PGx in their clinical practice	3 (2%)	29 (22%)	99 (76%)	0.6	0.3	0.3
In future, health provider must consult Pharmacists on PGx testing	3 (2%)	25 (19%)	103 (79%)	0.6	0.4	0.4
In future, providers must consult Pharmacists on therapy changes after PGx testing	4 (3%)	34 (26%)	93 (71%)	0.4	0.2	0.08
In future Pharmacists should use PGx tests for medication therapy management	3 (2%)	20 (15%)	107 (82%)	0.5	0.7	0.6
In future, Pharmacists should carry out PGx tests	6 (5%)	36 (27%)	89 (68%)	6.8	2.9	<0.0005
